# Acquired and Genetic Causes of Heart Disease Can Co-Exist in a Single Patient

**DOI:** 10.1016/j.jaccas.2025.103363

**Published:** 2025-03-05

**Authors:** Rafik Tadros, Paloma Jordà

**Affiliations:** a Cardiovascular Genetics Centre, Montreal Heart Institute and Faculty of Medicine, Université de Montréal, Montreal, Quebec, Canada; b Cardiovascular Genetics and Heart Failure Units, Cardiology Department, Hospital Universitari de Girona Dr. Josep Trueta, Girona, Spain. Cardiovascular Genetics Center, Institut d'Investigació Biomèdica Girona (IDIBGI), Girona, Spain

**Keywords:** cardiac arrhythmia, cardiomyopathy, genetics, sudden death

**Figure:**
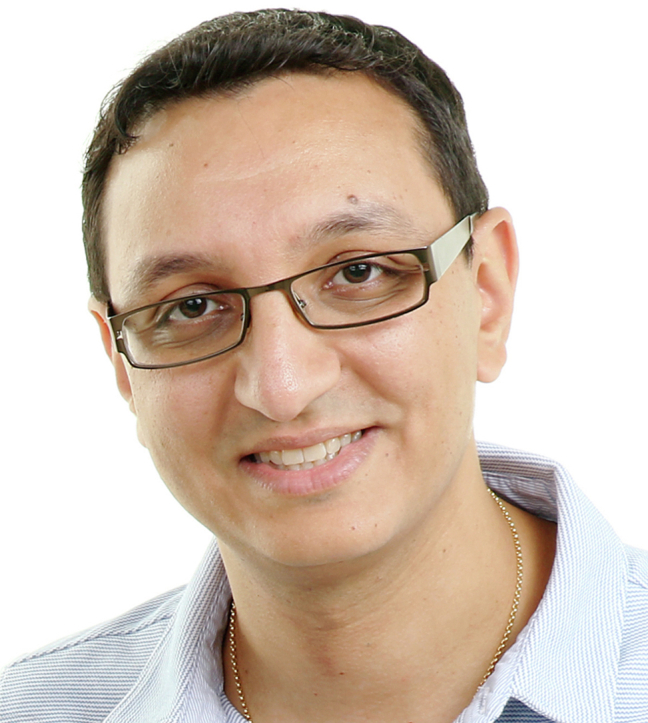

Traditionally, human diseases have been classified as either inherited or acquired. Medical trainees are instructed to make a differential diagnosis as part of their clinical reasoning, and the diagnostic workup is targeted towards identifying a single diagnosis/etiology. As cardiovascular clinicians, we often view acquired and genetic causes of heart disease as being mutually exclusive. Mounting data over the past decade have highlighted the contribution of monogenic culprits (ie, genetic mutations) in acquired heart diseases,^1^, ^2^, ^3^, ^4^ translating into changes in clinical practice with regards to genetic counselling and testing and family screening.^5^

In this issue of *JACC: Case Reports*, Ditaranto et al^6^ report a case of cocaine-induced ventricular tachycardia and a type I Brugada syndrome electrocardiogram (ECG) pattern. The patient died suddenly years later, also in the context of cocaine use. Because of the documented Brugada syndrome ECG, post-mortem genetic testing was performed and identified a likely pathogenic variant in *SCN5A*, the gene coding for the cardiac sodium channel and linked definitively to Brugada syndrome. Although the pathogenicity of the novel *SCN5A* variant may be challenged in the absence of functional studies and/or cosegregation analysis in the family, the case provides suggestive evidence that genetic factors may contribute to cocaine-induced malignant arrhythmia.

Although the yield of genetic testing in all cocaine-induced sudden cardiac arrest/death is likely to be low, the case suggests that patients with a cocaine-induced type I Brugada syndrome ECG should be referred for *SCN5A* testing. This can provide guidance for patient management and facilitate family screening.

The case of a genetic contribution to cocaine-induced electrical heart disease extends the list of acquired heart conditions with demonstrated monogenic contributions (Figure 1). In such conditions, genetic and acquired etiologies appearing in a differential diagnoses list are not mutually exclusive but can both contribute to disease expression. Importantly, identification of genetic etiologies coexisting with acquired causes have important implications for the prognosis of the individual and for screening of his relatives. In practice, systematic referral of all patients with acquired forms of cardiomyopathies, drug-induced long QT, or Brugada ECG for genetic testing may have a low yield. In systems with limited access to genetic testing, clinical judgement is warranted for proper selection of patients who are more likely to benefit from genetic testing. Features in the patient’s presentation and family history can point towards an underlying monogenic culprit. For instance, drug-induced torsades de pointes in a young patient with a single QT prolonging drug and mild QT prolongation following drug withdrawal is more likely to have a positive genetic test. Similarly, a young/middle-aged patient with asymmetric septal hypertrophy and only mild (grade I) hypertension is likely to have genetic hypertrophic cardiomyopathy. Finally, the presence of family history of certain cardiac symptoms/diseases will also be supportive of a monogenic contribution. Further studies on the yield and clinical impact of genetic testing in acquired conditions are needed to provide evidence-based guidance for management of the patient and their relatives.

**Figure 1:**
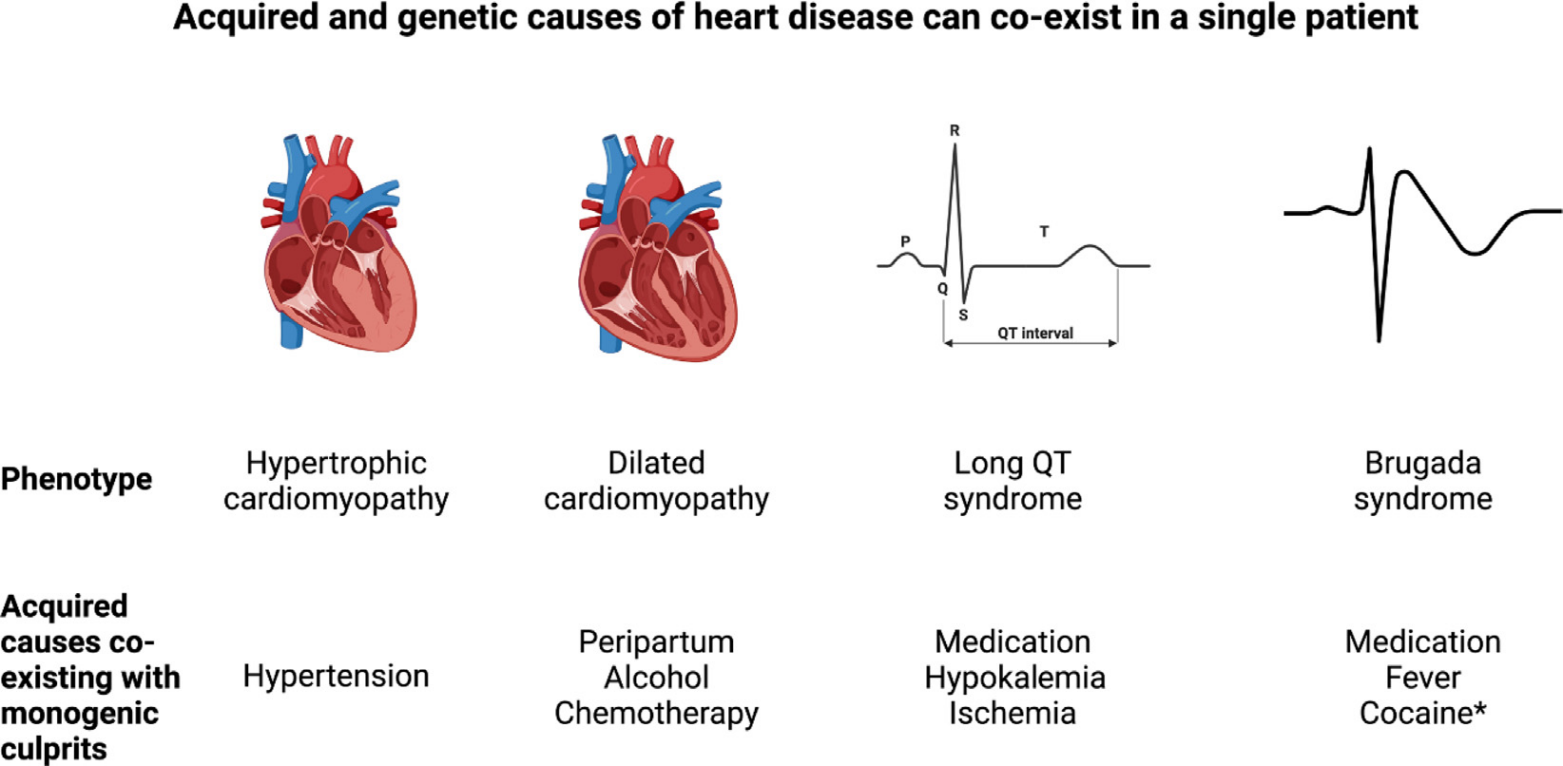
Acquired Conditions Causing Heritable Heart Diseases With Reported Monogenic Contributions Asterisk denotes the case reported in this issue of *JACC: Case Reports*. Figure drawn using BioRender.

## Funding Support and Author Disclosures

Dr Tadros has received research support and consulting fees from Bristol Myers Squibb. Dr Jordà has reported that she has no relationships relevant to the contents of this paper to disclose.
